# Integrated Phytochemical Profiling, UPLC-HRMS Characterization, and Bioactivity Evaluation of *Zingiber officinale* and *Piper nigrum*

**DOI:** 10.3390/ijms26167782

**Published:** 2025-08-12

**Authors:** Aicha Boubker, Abdelmoula El Ouardi, Taha El Kamli, Mohammed Kaicer, Faouzi Kichou, Khaoula Errafii, Adnane El Hamidi, Rachid Ben Aakame, Aicha Sifou

**Affiliations:** 1 Laboratory of Materials, Nanotechnologies and Environment, Faculty of Sciences, Mohammed V University in Rabat, BP: 1014, Ibn Battouta Avenue, Rabat 10000, Morocco; aicha.boubker@um5r.ac.ma (A.B.); a.elhamidi@um5r.ac.ma (A.E.H.); 2 Department of Microbiology of Water, Food and Environment, National Institute of Hygiene, BP: 769, Agdal, 27, Avenue Ibn Batouta, Rabat 10000, Morocco; abdoelouardi@yahoo.fr; 3 Laboratory of Anti-Doping Control, Hassan II Institute of Agronomic and Veterinary, BP: 6202, Madinat Al Irfane, Rabat 10101, Morocco; elkamlit@yahoo.fr; 4 Laboratory of Analysis, Geometry and Applications, Systems and Optimization, Ibn Tofail University, Campus of University, BP: 242, Kenitra 14000, Morocco; mohammed.kaicer@uit.ac.ma; 5 Department of Pathology and Veterinary Public Health, Hassan II Institute of Agronomic and Veterinary, BP: 6202, Madinat Al Irfane, Rabat 10101, Morocco; kichou.faouzi@gmail.com; 6 African Genom, University Mohammed VI Polytechnic Center, BP: 660, Hay Moulay Rachid, Benguerir 43150, Morocco; khaoula.errafii@um6p.ma; 7 Laboratory of Food Toxicology, National Institute of Hygiene, BP: 769, Agdal, 27, Avenue Ibn Batouta, Rabat 10000, Morocco; benakame@yahoo.fr

**Keywords:** phytochemical, antimicrobial activities, antioxidant activity, *Z. officinale*, *P. nigrum*, polyphenols, flavonoids, tannin, trace elements, UPLC-HRMS Orbitrap

## Abstract

The phytochemical profiles, antioxidant capacities, mineral composition, and antibacterial activities of *Zingiber officinale* (*Z. officinal*) and *Piper nigrum* (*P. nigrum*) were explored through aqueous, ethanolic, and methanolic extractions. The extracts were analyzed for polyphenols, flavonoids, and tannins, and their antioxidant potential was assessed using the DPPH assay. UPLC-HRMS identified major bioactive compounds, including 6-gingerol and shogaol in *Z. officinale*, and piperine and piperlonguminine in *P. nigrum*. Mineral analysis showed that *P. nigrum* was particularly rich in essential elements, including calcium (Ca), magnesium (Mg), and iron (Fe). In antibacterial testing, *P. nigrum* demonstrated wider zones of inhibition against *E. coli*, whereas *Z. officinale* was more active at lower concentrations, showing MICs as low as 3.91 µg/mL against *Salmonella* and *S. aureus*. PCA analysis revealed strong correlations between phenolic content and biological effects. These results underscore the potential of both spices as effective natural agents for use in food preservation and health-promoting applications.

## 1. Introduction

For centuries, medicinal plants have served as essential components of traditional healthcare systems, valued for their therapeutic and preventive benefits. In the context of the COVID-19 pandemic, renewed interest has emerged regarding their potential to support recovery, particularly due to their anti-inflammatory and antioxidant activities. These properties may help alleviate post-infection symptoms and support physiological homeostasis during the recovery period [1]. During the pandemic, spice consumption increased significantly in surveyed populations, including notable rises in the use of condiments and spices such as ginger and black pepper during lockdowns [2,3]. The increasing global interest in spice-derived compounds has recently stimulated advances in plant genomics, aimed at improving both the sustainability and efficiency of their cultivation [4].

Ginger (*Zingiber officinale*) is one of the most widely used spices, appreciated for its dual role in both culinary applications and traditional medicine. In Moroccan ethnomedicine, it is particularly recognized for its immunomodulatory and anti-inflammatory properties, notably in the management of immune-related disorders such as rheumatoid arthritis [5]. Rhizomes of the *Zingiber* genus, widely recognized for their culinary and therapeutic applications, exhibit notable antibacterial activity, largely attributed to their richness in bioactive phytochemicals [6]. Recent research highlights that *Z. officinale* contains high levels of 6-gingerol and 6-shogaol, compounds with proven antioxidant, anti-inflammatory, and anticancer effects [7]. Similarly, black pepper (*Piper nigrum*) is rich in bioactive phytochemicals, particularly piperine and essential oil constituents, which play a key role in its antibacterial and anti-virulence activities [8].

In Moroccan cuisine, the connection between food and health is deeply rooted, as reflected in the frequent use of *Z. officinale* both as a medicinal remedy and as a flavoring agent in traditional dishes [9].

Similarly, *P. nigrum* is widely used not only as a culinary spice but also as a medicinal agent in pharmaceutical preparations and food preservation. It is valued for its antibacterial and antioxidant properties, as well as its ability to enhance the bioavailability of other compounds [10]. Beyond its culinary applications, *P. nigrum* has traditionally been used for its potential cognitive benefits. Recent clinical studies suggest that it may aid in the management of Alzheimer’s disease by reducing neuroinflammation and supporting memory function [11]. Widely appreciated for its dual culinary and therapeutic applications, *P. nigrum* owes much of its medicinal value to piperine, its principal bioactive constituent, which has been shown to exert cardioprotective effects, notably through lipid regulation, blood pressure reduction, and antioxidant activity [12].

*P. nigrum* holds an important place in Moroccan cuisine, where it is commonly incorporated into traditional dishes and spice blends such as *Ras el Hanout*. Beyond its aromatic contribution, it is also recognized for its antibacterial properties and potential role in food preservation [13]. Its essential oil has demonstrated strong antibacterial and antibiofilm activities, making *P. nigrum* a promising natural preservative for enhancing food safety and extending shelf life [14].

Although *Z. officinale* and *P. nigrum* are generally considered safe at culinary doses, their excessive consumption may lead to adverse effects. High doses of ginger have been linked to digestive discomfort and potential interactions with medications, particularly anticoagulants [15]. Similarly, large amounts of black pepper or piperine may irritate the gastrointestinal tract and affect drug metabolism [16]. These considerations highlight the importance of evaluating both the therapeutic benefits and potential risks associated with their use.

Using a multi-analytical approach, we thoroughly assessed two popular spices, *Z. officinale* and *P. nigrum,* to investigate their phytochemical composition, antioxidant potential, bioactive compounds, mineral content, and antibacterial activity against common foodborne pathogens. In particular, UPLC-HRMS was employed to identify and characterize the major bioactive compounds to elucidate their potential as natural antibacterial agents and contributors to health promotion. We used Principal Component Analysis (PCA) to evaluate correlations between important metrics and further distinguish their distinct phytochemical profiles. The scientific validation of their pharmaceutical relevance was reinforced by the application of multivariate statistical techniques, which provided deeper insight into their distinct and shared biochemical characteristics. A thorough examination of these bioactive compounds will advance understanding of their functional characteristics, thereby supporting their potential applications in natural therapeutics, food preservation, and the development of functional foods.

## 2. Results

### 2.1. Total TPC, TFC, TCT, and Antioxidant Activities

Table 1 shows that *Z. officinale* exhibited a higher polyphenol content when extracted with ethanol (75.694 µg GAE/mg extract). In constructs followed by methanol (54.523 µg GAE/mg extract), the aqueous extract presented the lowest concentration (4.105 µg GAE/mg extract). In comparison, *P. nigrum* showed lower total polyphenol contents, with the highest values observed in the ethanolic and methanolic extracts. According to [17], the total phenolic content of *P. nigrum* ethanolic and methanolic seed extracts reached 52.6 ± 3.1 mg GAE/g and 41.5 ± 3.4 mg GAE/g, respectively, suggesting that the choice of extraction solvent significantly influences the recovery of bioactive compounds.

Regarding flavonoid content, *Z. officinale* also demonstrated significantly higher levels, with the highest concentration in the ethanolic extract, followed by the methanolic and aqueous extracts. This pattern aligns with the findings of [18], who reported flavonoid contents of 609.66 mg QE/g and 563.10 mg QE/g in ethanolic and methanolic extracts, respectively. Conversely, *P. nigrum* exhibited very low flavonoid concentrations, ranging from 2.086 to 5.617 µg QE/mg extract. Although the polyphenol and flavonoid contents of *P. nigrum* reported in our study are lower than those cited in [17,18], these discrepancies may be due to differences in extraction protocols, plant origin, or unit expression. Nonetheless, the observed antioxidant activity suggests a significant contribution of *P. nigrum* bioactive constituents, particularly piperine.

*P. nigrum* is much richer in tannins than *Z. officinale*, particularly in its ethanolic extract (21.17 µg EC/mg extract), compared with just 2.407 µg EC/mg extract in the ethanolic extract of ginger. This observation is consistent with findings by Akullo et al., who reported that the tannin content in the ethanolic extract of local ginger reached 42.36 mg catechin equivalent per 100 g, which is markedly lower than the levels observed in *P. nigrum* under similar extraction conditions [19].

*P. nigrum* has been extensively studied for its role in metabolic regulation and antioxidant defense, largely attributed to its principal bioactive compound, piperine. This alkaloid has shown promising effects in mitigating inflammatory responses and improving lipid metabolism in both clinical and preclinical investigations [20]. Moreover, methanolic extracts of P. nigrum have exhibited strong free radical scavenging activity in DPPH assays, indicating their potential to neutralize reactive oxygen species and prevent oxidative stress-induced cellular damage [21]. In our study, we found ascorbic acid at a concentration of 4.12 μg/mL, highlighting its contribution to the extract’s overall antioxidant activity.

### 2.2. Determination of Bioactive Molecules by UPLC-HRMS Orbitrap

Significant variations in the profiles of bioactive compounds, which are correlated with the biological activities of *Z. officinale* and *P. nigrum*, were found by comparing their phytochemical analyses. Phenolic substances, including 6-gingerol, shogaol, cinnamic acid, and 5-carboxyvanillic acid, which exhibit significant antioxidant activity and potential anticancer effects, were discovered to be especially abundant in *Z. officinale*. These findings are consistent with other research showing the phenolics generated from ginger can scavenge radicals and may have a function in preventing disease [22,23,24]. However, the inclusion of alkaloids and terpenoids, such as trans-geranic acid, piperine, piperanine, and piperlonguminine, which had significant antibacterial and anti-inflammatory qualities, was what distinguished *P. nigrum* [25,26]. The historic use of *P. nigrum* as a natural preservative in food systems is supported by the potent antibacterial properties of piperine and piperanine against harmful microbes [27,28]. Moreover, bergenin and piperlonguminine were found to be important anti-inflammatory substances, which perhaps help explain why *P. nigrum* has therapeutic benefits for reducing inflammatory conditions [29,30]. Recent studies have highlighted the role of *P. nigrum* bioactive compounds, especially piperamides, in modulating inflammation and oxidative stress. The authors of [31] demonstrated that guineensine and related compounds exhibit strong inhibition of anandamide cellular uptake, indirectly modulating the endocannabinoid system and offering potential therapeutic benefits.

Although our study focused on bioactive molecules, antioxidant capacity, mineral profiling, and antibacterial effects, we did not assess the proximate nutrient composition of *Z. officinale* and *P. nigrum* (such as carbohydrates, proteins, lipids, and fibers) Table 2. This decision was based on our objective to explore their functional and therapeutic potential. However, the recent literature already provides detailed nutritional data for these spices, as in [32,33]. Future investigations may incorporate nutrient profiling to complement bioactivity studies and provide a more comprehensive evaluation of their health benefits.

### 2.3. Mineral Metal Contents in the Plants

The mineral profiling of *Z. officinale* and *P. nigrum* revealed, in Table 3, distinct differences in their elemental content, reflecting their unique nutritional and therapeutic potential. Overall, *P. nigrum* exhibited significantly higher concentrations of both macro- and microelements compared to *Z. officinale*, indicating a more mineral-rich profile. The mineral elements analyzed (Ca, Mg, Fe, Zn, etc.) were selected based on their documented nutritional relevance in spices, as highlighted by [48], who emphasized their contribution to essential physiological functions such as immunity and enzymatic activity.

Calcium (Ca) content was markedly higher in *P. nigrum* (4.160 mg/g dry matter) than in *Z. officinale* (1.430 mg/g), consistent with previous reports highlighting *P. nigrum* as a good source of calcium, important for bone health and neuromuscular function [49]. Similarly, *P. nigrum* showed elevated levels of magnesium (1.923 mg/g) and potassium (0.589 mg/g) in our study. These findings are consistent with those reported by [50], who identified even higher concentrations of these minerals in *P. nigrum* leaves, particularly potassium (164.45 mg/100 g) and magnesium (94.75 mg/100 g), confirming the nutritional richness of this spice in essential electrolytes involved in muscle contraction and metabolic regulation.

Although *Z. officinale* exhibited a slightly higher level of magnesium (2.371 mg/g), it contained significantly less potassium (0.751 mg/g), suggesting a different balance in mineral utilization. Ref. [32] reported lower magnesium levels (1.795 mg/g) but higher potassium content (2.935 mg/g) in ginger cultivated in the Himalayan region of India, highlighting the influence of environmental and agronomic conditions on mineral composition.

In terms of trace elements, *P. nigrum* had substantially higher levels of iron (Fe) (4.030 mg/g), zinc (Zn) (0.650 mg/g), manganese (Mn) (1.760 mg/g), and copper (Cu) (0.300 mg/g), all of which are essential cofactors in enzymatic systems and immune regulation. Our results showed higher concentrations compared to [51]. In contrast, *Z. officinale* displayed considerably lower concentrations of these elements, suggesting a more limited contribution to trace mineral intake.

The presence of essential elements such as Na and B highlights the nutritional value of *Z. officinale* and *P. nigrum*. As emphasized by [52], comprehensive elemental analysis remains crucial due to global variability in spice composition.

The mineral richness of culinary spices such as *P. nigrum* and *Z. officinale*, particularly in calcium, potassium, and sodium, has been consistently reported across studies and is increasingly linked to their health-promoting effects. These essential elements contribute to key physiological processes, including immune modulation, enzymatic activation, and antioxidant defense. According to [53], black pepper exhibits a superior mineral profile compared to other common spices, while [54] highlights its bioactive potential concerning selenium and flavonoid content.

In everyday culinary use, these spices are usually added in small quantities—often less than 1 to 5 g per day. At such doses, their contribution to daily mineral requirements remains limited. For example, a 5 g portion of turmeric (another spice with a similar mineral profile) can provide over 20% of the recommended daily intake for iron and manganese, but contributes less than 5% for other minerals like calcium or potassium [55,56]. While spices are not primary sources of dietary minerals, their frequent and widespread use may help complement overall nutrient intake, particularly in diets that are marginally deficient.

### 2.4. Antibacterial Activity

The in vitro antimicrobial activity of *Z. officinale* and *P. nigrum* was evaluated using agar diffusion and broth microdilution assays. As shown in Table 4, *P. nigrum* demonstrated broader zones of inhibition across all tested strains, reaching 15.00 mm against *E. coli*, 12.50 mm against *Salmonella typhimurium*, and 14.00 mm against *Staphylococcus aureus* at a concentration of 100 mg/mL. *Z. officinale* exhibited comparable inhibition zones, with 14.00 mm against *E. coli* and 12.00 mm against *S. aureus*, and showed activity against Salmonella only at the highest concentration tested. Their effects were compared to those of standard antibiotics. As expected, ciprofloxacin, gentamicin, and oxacillin produced significantly larger inhibition zones, between 23 and 28 mm. However, both *Z. officinale* and *P. nigrum* showed clear antimicrobial activity at high concentrations, particularly against *E. coli* and *S. aureus*. Although their effects were more modest than those of the antibiotics, the observed inhibition zones (ranging from 12 to 15 mm) remain within the expected range for crude plant extracts.

However, MIC results (Table 5) revealed a stronger bacteriostatic effect for *Z. officinale*, with remarkably low MIC values of 3.91 µg/mL against both *S. aureus* and *Salmonella*. In comparison, *P. nigrum* showed MICs of 62.5 µg/mL and 31.25 µg/mL for the same strains. These results indicate that while *P. nigrum* offers a broader antimicrobial spectrum at high doses, *Z. officinale* is more effective at lower concentrations.

To contextualize these findings, MIC values of standard antibiotics used as positive controls were also assessed: *Ciprofloxacin* exhibited MICs ranging from 0.015 to 0.03 µg/mL against *E. coli*, *Gentamicin* ranged from 1 to 5 µg/mL against *Salmonella*, and *Oxacillin* showed MICs between 0.25 and 2 µg/mL against *S. aureus.* As expected, these antibiotics displayed far greater potency. Nevertheless, the detectable activity of *Z. officinale* and *P. nigrum*, especially at sub-milligram concentrations, remains relevant in the context of natural antimicrobial alternatives.

These findings align with previous studies attributing *P. nigrum*’s activity to piperine, known for its bactericidal and antibiofilm effects [57], and supports its efficacy, as demonstrated in nanoparticle-mediated formulations [58]. Additionally, thermal resistance data reported by [59] reinforce the relevance of *P. nigrum* in food safety applications.

The antibacterial evaluation of *Z. officinale* revealed promising results, with inhibition zones reaching 14 mm against *E. coli* and 12 mm against *S. aureus* at 100 mg/mL. Remarkably low MIC values were observed (3.91 µg/mL for *S. aureus* and *Salmonella*), indicating strong bacteriostatic potential. These findings are consistent with previous studies. Ref. [60] reported an MIC90 of 13.27 mg/mL against *Staphylococcus* spp., while [61] confirmed strong antibacterial effects linked to polyphenol content. Furthermore, Ref. [62] reported comparable inhibition zones but higher MICs, which suggests the high potency of our aqueous extract. The mode of action may involve membrane disruption, as described by [63], reinforcing the therapeutic relevance of ginger in controlling pathogenic bacteria.

Beyond their demonstrated antibacterial efficacy, *Z. officinale* and *P. nigrum* also offer promising applications as natural food preservatives and immune-enhancing agents. Recent reviews have highlighted the antimicrobial potential of spice-derived compounds, particularly in extending the shelf-life of meat and preventing foodborne illnesses, while avoiding the toxic effects of synthetic additives [64,65]. Additionally, both spices are core components of traditional herbal formulations, such as Ayush Kwath, recommended during the COVID-19 pandemic for their immunomodulatory, antiviral, and anti-inflammatory properties [66,67]. These multifaceted properties reinforce the role of culinary spices not only as therapeutic agents but also as sustainable, health-promoting food additives.

### 2.5. Correlation Matrix

**Figure 1:**
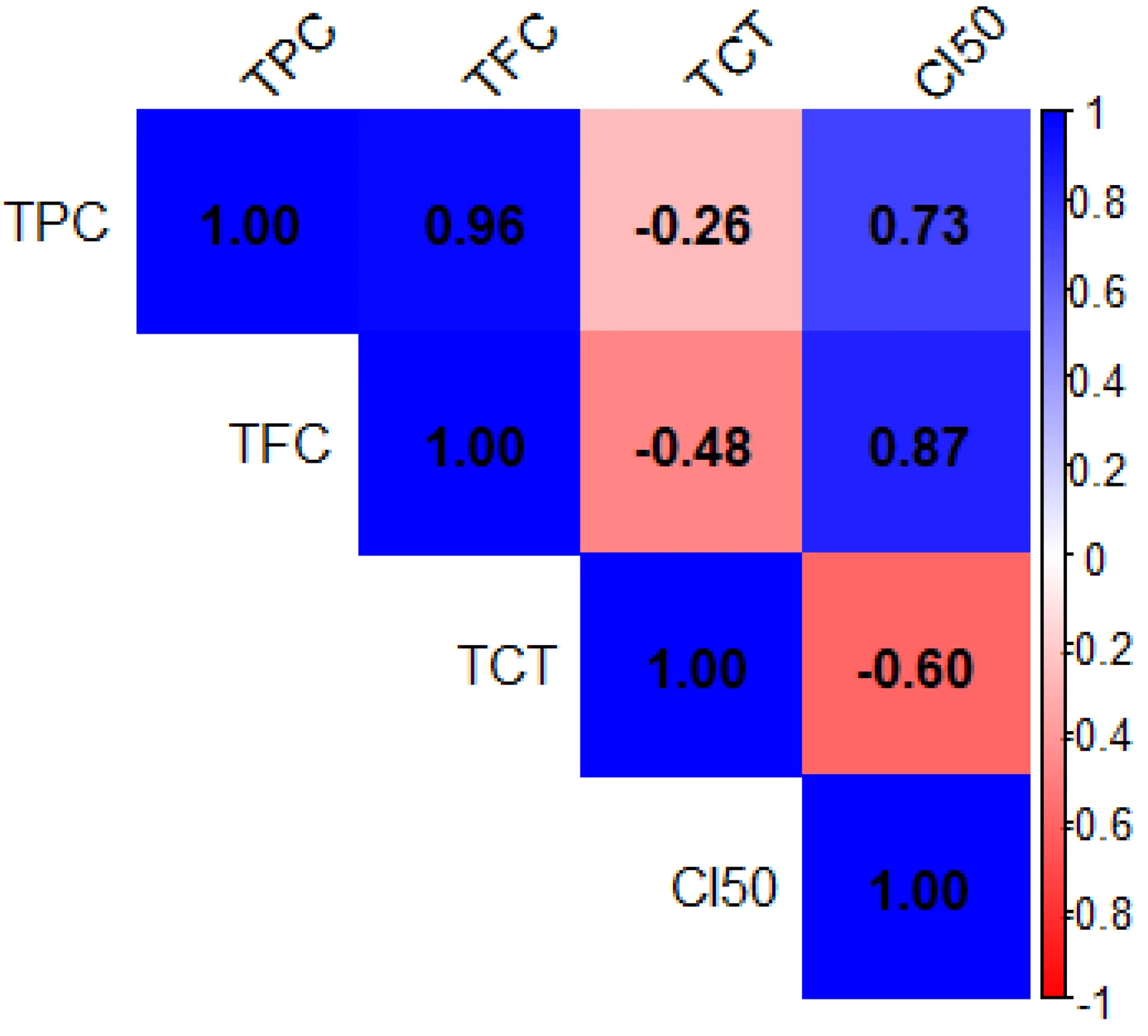
Correlation matrix of total phenolic content (TPC), total flavonoid content (TFC), total condensed tannins (TCT), and antioxidant activity (CI_50_) in *Z. officinale* and *P. nigrum* extracts. The axes (X and Y) represent the variables analyzed. The color scale to the right shows the strength and direction of correlations: blue indicates strong positive correlation (r ≈ +1), red indicates a strong negative correlation (r ≈ −1), and white indicates a weak or no correlation (r ≈ 0). Numerical values inside the cells correspond to Pearson correlation coefficients.

The heatmap of Pearson correlation coefficients (Figure 1) illustrates the relationships between the total phenolic content (TPC), total flavonoid content (TFC), total condensed tannins (TCT), and antioxidant activity (CI_50_) in *Z. officinale* and *P. nigrum* extracts. The color scale represents the strength and direction of these correlations, with blue indicating positive correlations and red representing negative correlations. The data revealed a very strong positive correlation between total polyphenol content (TPC) and total flavonoid content (TFC) (r = 0.96), indicating that these two classes of compounds are often co-extracted and may be present in similar proportions across samples. A similar trend was observed between TFC and antioxidant activity, as expressed by CI_50_ values (r = 0.87), which suggests that flavonoids contribute meaningfully to the extracts’ radical scavenging potential.

**Figure 2:**
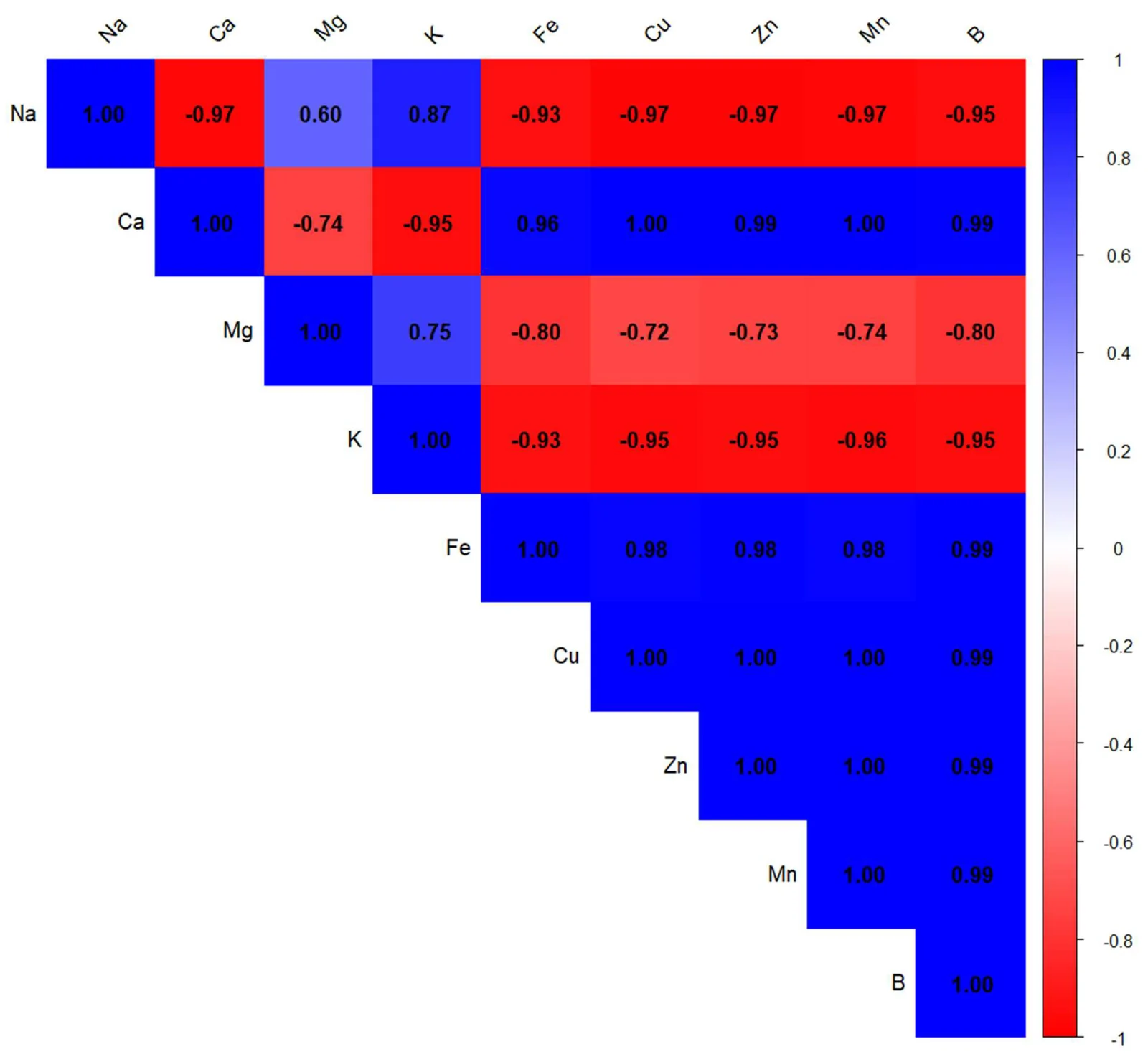
Correlation matrix of mineral composition in *Z. officinale* and *P. nigrum* extracts. The horizontal and vertical axes indicate the mineral variables included in the analysis. Each cell displays the Pearson correlation coefficient between two minerals. The color scale on the right represents the strength and direction of the correlation: blue indicates a strong positive correlation (r ≈ +1), red indicates a strong negative correlation (r ≈ −1), and white indicates a weak or no correlation (r ≈ 0).

Conversely, condensed tannins (TCT) displayed a moderate inverse relationship with both TFC (r = −0.48) and CI_50_ (r = −0.60), implying that high levels of tannins might not directly enhance antioxidant performance. Notably, TPC was also moderately correlated with CI_50_ (r = 0.73), reinforcing the idea that polyphenols as a group are key contributors to antioxidant defense. These observations underscore the importance of flavonoids in antioxidant mechanisms, while also suggesting that not all phenolic subclasses contribute equally to this activity. The heatmap of Pearson correlation coefficients (Figure 2) illustrates the relationships between the mineral elements present in *Z. officinale* and *P. nigrum* extracts. The color scale represents the strength and direction of these correlations, with dark blue indicating strong positive correlations (close to +1) and red representing strong negative correlations (close to 1).

A strong inverse correlation was observed between sodium and several trace elements (r ≈ −0.95), suggesting possible antagonistic interactions that may impact their bioavailability and functional roles within the extracts. Similarly, potassium also showed strong negative correlations with iron (r = −0.93), copper (r = −0.95), zinc (r = −0.95), manganese (r = −0.96), and boron (r = −0.95). This pattern implies that elevated potassium levels might be associated with reduced concentrations of these micronutrients, potentially disrupting mineral equilibrium. 

Magnesium, in contrast, exhibited a moderate positive correlation with sodium (r = 0.60), while showing negative correlations with iron (r = −0.80), copper (r = −0.72), zinc (r = −0.73), and manganese (r = −0.74). These findings suggest that increased magnesium content could limit the availability of certain trace elements, possibly due to competition for absorption sites or complexation phenomena within the extract matrix. The observed correlations among mineral elements point to specific patterns of interaction and uptake in *Z. officinale* and *P. nigrum*. Such mineral associations may influence the extracts’ functional properties and contribute to their overall physiological effects. Our findings align well with previous research showing strong links between polyphenol levels and antioxidant activity. Ref. [68] reported very high correlations—often above 0.95—between total phenolics and flavonoids in various spices and herbs. These compounds were also strongly associated with antioxidant effects, measured through tests like DPPH, ABTS, and FRAP. Similarly, Ref. [69] found a strong linear relationship (R^2^ = 0.89–0.97) between antioxidant activity and the content of polyphenols, flavonoids, and tannins in mace (*Myristica fragrans*). These studies support the idea that the antioxidant potential of plant extracts is closely tied to their content of bioactive phenolic compounds.

### 2.6. Principal Component Analysis

**Figure 3:**
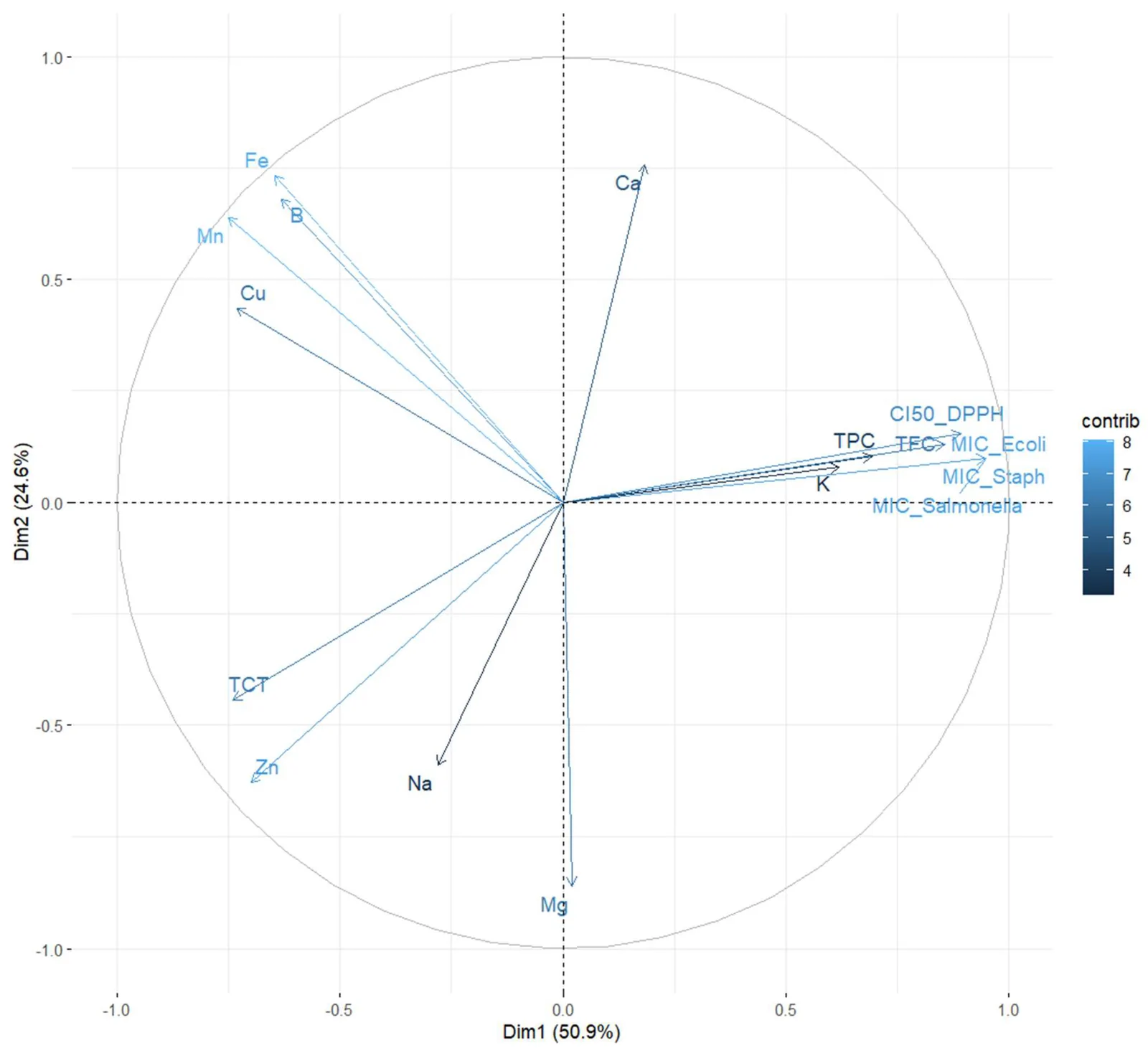
Principal Component Analysis (PCA) biplot illustrating the relationships among antioxidant markers, antimicrobial activity, and mineral content in *Z. officinale* and *P. nigrum* extracts. The horizontal axis (Dim1: 50.9%) reflects the **Antioxidant–Antimicrobial Axis**, strongly influenced by TPC, TFC, CI_50_, and MIC values. The vertical axis (Dim2: 24.6%) reflects the **Mineral Composition Axis**, capturing variance in elements like Fe, Mn, Cu, and B. Vectors represent the variable contributions to each component, and the proximity between them indicates the correlation strength.

The plane defined by Dim1 (50.9%) and Dim2 (24.6%), together explaining 75.5% of the total variance, was used to represent the distribution of variables in the PCA biplot (Figure 3). The vectors illustrate the influence of each variable on the two principal components, with their coloration reflecting the strength of their contribution according to the “contrib” scale.

Variables such as TPC, TFC, CI_50__DPPH, and MIC_*E. coli*, MIC_*Salmonella*, and MIC_*Staph* show a strong positive loading on Dim1 (right side of the plot), indicating a clear association between antioxidant capacity and antibacterial activity. Their clustering on the graph suggests that extracts rich in polyphenols and flavonoids tend to exhibit enhanced antimicrobial and antioxidant properties. These observations are consistent with earlier reports suggesting that these bioactive compounds may act synergistically to enhance the biological efficacy of plant extracts.

In contrast, sodium, magnesium, and zinc, positioned on the left side of Dim1, exhibit an opposing trend, indicating a negative association with antioxidant and antimicrobial activities. This inverse relationship may reflect their potential role in modulating chemical interactions within the extract matrix, possibly diminishing overall bioactivity.

Along Dim2, iron, manganese, copper, and boron display strong positive contributions, suggesting their influence may extend beyond direct biological effects (e.g., CI_50_ or MIC), potentially impacting structural properties or influencing the bioavailability of other constituents.

Notably, calcium is located away from the main cluster of bioactive markers, implying that it may serve a distinct function in the chemical composition of the extracts, without directly contributing to their antioxidant or antibacterial properties. The total condensed tannins (TCT) appear negatively correlated with biological activities and are located in the lower left quadrant of the PCA plot. This positioning suggests that, unlike polyphenols and flavonoids, tannins may play a limited or even inverse role in contributing to the antioxidant and antibacterial properties of the extracts. Potassium (K) also displays a mild opposition to the principal bioactive compounds, implying that elevated potassium levels might be associated with reduced antimicrobial or antioxidant efficiency. This could be attributed to its primary function in cellular processes rather than direct involvement in bioactivity.

Our PCA results clearly showed that extracts rich in polyphenols and flavonoids tend to have stronger antioxidant and antibacterial effects. This pattern has also been seen in other studies, where similar compounds grouped closely with bioactivity markers in PCA plots [70,71]. These findings further support the idea that these natural compounds are key drivers of the health benefits found in plant-based extracts.

Altogether, these results offer meaningful insights into the chemical–biological interplay within the plant matrices studied, highlighting the dominant influence of polyphenols and flavonoids in driving antioxidant and antimicrobial performance.

## 3. Materials and Methods

### 3.1. Plant Material

In March 2024, dried rhizomes of *Z. officinale* and dried fruits of *P. nigrum* were purchased from a local herbalist in Rabat, Morocco (33°59′56.071″ N, 6°50′57.055″ W). The herbalist acted solely as a reseller and was not involved in the cultivation process. According to the vendor, both plant materials were harvested at full maturity during the same agricultural season (early 2024), ensuring consistency in their phytochemical profiles. The selection of rhizomes for *Z. officinale* and fruits for *P. nigrum* was based on their traditional use in Moroccan ethnopharmacology, where these specific plant parts are recognized for their high content of bioactive compounds and therapeutic relevance.

Upon acquisition, the materials were transported to the National Institute of Hygiene, where they were stored in labeled cardboard boxes (approximately 100 g per sample) under dry, ambient conditions. Before extraction, each plant sample was finely ground into a homogeneous powder using a laboratory-grade blender (Model FG-109, Elite, locally sourced in Morocco) to facilitate solvent penetration and ensure reproducibility in downstream analyses.

### 3.2. Preparation of Extracts

To investigate the biochemical characteristics of the selected plant materials, each part was first finely ground into a homogeneous powder. Then, 10 g of the powdered material from each plant was extracted with 100 mL of either ethanol or methanol (≥99.8% purity; Sigma-Aldrich, Merck, Darmstadt, Germany) or distilled water produced in-house using a Milli-Q^®^ purification system (Millipore, Billerica, MA, USA). The extraction was performed by maceration at room temperature for 24 h, following the protocol described by [72]. The mixtures were filtered through Whatman toughened filter paper (AHLESS, round, 125 mm diameter, Cytiva brand, United Kingdom), and the solvents were evaporated under reduced pressure using a rotary evaporator (model EVA180, IBX Instruments, Barcelona, Spain).

The resulting extracts were semi-viscous (not fully dried) and were stored in sterile Eppendorf tubes at 4 °C until analysis. For biochemical assays such as total polyphenol, flavonoid, condensed tannin, and antioxidant activity, the concentrated extracts were diluted appropriately. For the antibacterial evaluation, only the aqueous extracts were used and re-dissolved in sterile distilled water before testing.

### 3.3. Determination of Total Polyphenol Content (TPC)

The Folin–Ciocalteu (FC) test was used to measure the total polyphenol content in the *Z. officinale* and *P. nigrum* extracts, as explained by [73]. Methanolic gallic acid standards ranging from 0 to 200 μg/mL, made from a 0.5 g/L stock solution, were used to create a calibration curve. A volume of 200 μL of each plant extract was combined with 1 mL of 10% FC reagent, and the mixture was incubated for 20 min in the dark at a regulated temperature of 25 °C to ensure optimal reaction conditions. Subsequently, a blank and 800 μL of 7.5% (*w*/*v*) sodium carbonate solution were added to the reaction mixture. After thorough mixing, the solution was incubated for three hours at a controlled temperature in the dark to ensure complete color development. Absorbance was then measured at 765 nm using a UV–visible spectrophotometer (Peak Instrument C-7200A, Houston, TX, USA). Quantification was performed using a gallic acid reference curve, and results were expressed as µg GAE per mg of dry plant matter.

### 3.4. Determination of Total Flavonoid Content (TFC)

The total flavonoid content in the extracts of *Z. officinale* and *P. nigrum* was determined using a colorimetric method, as described by [74]. In brief, 1.25 mL of distilled water, 0.075 mL of 5% (*w*/*v*) aqueous sodium nitrite (NaNO_2_), and 0.25 mL of plant extract were mixed. After 5 min, 0.15 mL of 10% (*w*/*v*) aluminum chloride (AlCl_3_) solution was added. Following a further 6 min incubation, 0.5 mL of 1 M sodium hydroxide (NaOH) was introduced into the reaction mixture. The final solution was allowed to incubate for 30 min, after which the absorbance was measured at 510 nm using a UV–visible spectrophotometer (Peak Instrument C-7200A). The flavonoid content was expressed as micrograms of quercetin equivalent per milligram of dry plant material (µg QE/mg), based on a quercetin standard calibration curve ranging from 5 to 60 µg/mL.

### 3.5. Determination of Total Condensed Tannins (TCT)

The concentration of condensed tannins in the extracts of *Z. officinale* and *P. nigrum* was assessed using the vanillin assay, following the protocol described by [73]. Briefly, 50 μL of each plant extract was mixed with 1.5 mL of a 4% vanillin solution prepared in methanol. Then, 750 μL of concentrated hydrochloric acid (HCl) was added, with a blank included for reference. The reaction mixture was left to stand at room temperature for 20 min. Absorbance was then measured at 500 nm using a UV–visible spectrophotometer (Peak Instrument C-7200A). Quantification was performed using a standard curve of catechin, prepared over the range of 200 to 1000 µg/mL. Results were expressed as micrograms of catechin equivalents per milligram of dry plant material.

### 3.6. Antioxidant Activity

The antioxidant capacity of the plant extracts was evaluated based on their ability to scavenge the DPPH (2,2-diphenyl-1-picrylhydrazyl) free radical, following the method described by [75]. In this assay, 0.5 mL of a 0.2 mM DPPH solution prepared in ethanol was mixed with 2.5 mL of the ethanolic plant extract at an appropriate dilution. The mixture was stirred thoroughly and incubated in the dark for 30 min, alongside a blank. After incubation, the absorbance was recorded at 517 nm using a UV–visible spectrophotometer. Ascorbic acid was used as a positive control due to its well-documented antioxidant properties. The percentage of DPPH radical scavenging activity was calculated using the following formula:

%Inhibition = [(Abs _Control_ − Abs _test_)/Abs _test_] * 100

### 3.7. Instrument and Chromatography Conditions

The chromatographic analysis was conducted using a Thermo Fisher Vanquish LC system, comprising a binary pump, an autosampler, and a column oven. Separation was achieved on a Hypersil GOLD C18 column (150 × 2.1 mm, 3 µm particle size), coupled to a high-resolution accurate-mass spectrometer (Orbitrap Exploris 120, Thermo Scientific, Waltham, MA, USA). The mobile phase consisted of solvent A (methanol with 0.1% formic acid) and solvent B (water with 0.1% formic acid). The gradient elution was carried out according to the following program: the run began with a mixture of 70% solvent A and 30% solvent B from 0.00 to 1.00 min. This was followed by a transition to 100% solvent B, maintained from 1.01 to 20.00 min. The composition then shifted to 55% solvent A and 45% solvent B from 20.01 to 25.00 min. Finally, the initial conditions of 70% solvent A and 30% solvent B were re-established and held from 25.01 to 40.00 min. The flow rate was maintained at 0.30 mL/min, the column temperature was set to 30 °C, and the injection volume was 3 µL, following the procedure reported by [76].

### 3.8. Elemental Determination

Mineral content was assessed following a dry ashing method. Precisely 10 g of dried plant powder was incinerated in a programmable muffle furnace, gradually increasing the temperature from 100 °C to 450 °C over 7 h. After cooling, the resulting ash was moistened with 3 mL of distilled water and evaporated on a hot plate. The sample was then subjected to a second incineration step, beginning at 200 °C and ramping up to 450 °C for 2 h, following the addition of 5 mL of hydrochloric acid. After a final evaporation step, the ash was dissolved in 10 mL of 0.1 mol/L nitric acid, under the protocol recommended in reference [77]. The quantification of mineral elements was performed using a Varian AA240 Atomic Absorption Spectrometer, equipped with a Graphite Furnace (AA240 Z) and an autosampler. The elements analyzed included potassium (K), calcium (Ca), magnesium (Mg), manganese (Mn), copper (Cu), iron (Fe), zinc (Zn), boron (B), and sodium (Na).

### 3.9. Antibacterial Activities

#### 3.9.1. Agar Diffusion Test

The antibacterial properties of the aqueous extracts of *Z. officinale* and *P. nigrum* were evaluated using the disk diffusion method. Standard bacterial strains—*Escherichia coli* (ATCC 25922), *Salmonella typhimurium* (ATCC 14028), and *Staphylococcus aureus* (ATCC 25923)—were first cultured on nutrient agar and incubated at 37 °C for 24 h. Colonies were then harvested and suspended in sterile 0.9% NaCl solution to achieve a turbidity equivalent to 0.5 McFarland standard, corresponding to approximately 1 × 10^8^ CFU/mL. Sterile blank discs (6 mm in diameter) were impregnated with 10 µL of each extract and placed on Müller–Hinton agar plates previously inoculated with the test bacteria. Antibiotic discs were used as positive controls: ciprofloxacin (5 µg, Oxoid, Waltham, MA, USA) for *E. coli*, gentamicin (10 µg, Oxoid, Waltham, MA, USA) for *S. typhimurium*, and oxacillin (1 µg, Oxoid, Waltham, MA, USA) for *S. aureus*. Discs soaked in distilled water served as negative controls. All plates were incubated at 37 °C for 24 h. The procedure was carried out following the guidelines of the Clinical and Laboratory Standards Institute [78].

#### 3.9.2. Determination of Minimum Inhibitory Concentration of the Extract

The minimum inhibitory concentration (MIC) of the plant extracts was determined using a broth microdilution method. Dried extracts were first dissolved in distilled water, and 100 µL of Brain Heart Infusion (BHI) medium was dispensed into the wells of a sterile 96-well microplate. Each well then received 100 µL of the test extract at an initial concentration of 10,000 µg/mL. A series of two-fold serial dilutions was performed to obtain the following final concentrations: 5000, 2500, 1250, 625, 312.5, 156.2, 78.1, 39.0, and 19.5 µg/mL. A bacterial suspension adjusted to 10^8^ CFU/mL was prepared from a 24 h culture, and 10 µL of this suspension was inoculated into each well. Positive controls included ciprofloxacin (Sigma-Aldrich, Merck, Darmstadt, Germany, CAS: 93107-08-5), gentamicin sulfate (CAS: 1405-41-0), and oxacillin sodium salt monohydrate (CAS: 7240-38-2), while negative controls received no extract or antibiotic. Plates were incubated at 37 °C for 24 h. Following incubation, 10 µL of an MTT solution (3-(4,5-dimethylthiazol-2-yl)-2,5-diphenyltetrazolium bromide, 0.4 mg/mL in saline) was added to each well, and the plates were further incubated at 37 °C for 10 to 30 min. The protocol adhered to the guidelines of [79].

### 3.10. Statistical Data Analysis

Statistical analyses were conducted using RStudio (version 2024.06.2 + 492). The parameters analyzed included total polyphenol content (TPC), total flavonoid content (TFC), total condensed tannins (TCT), and CI_50_ values. Differences among these variables were assessed through analysis of variance (ANOVA), with results considered statistically significant at *p* < 0.05. Given previous studies highlighting the effectiveness of ethanol as an extraction solvent for phenolic compounds and antioxidant activity, only ethanol-based extracts were selected for further analysis. To explore the relationships among the measured variables and ethanol extracts, Principal Component Analysis (PCA) was performed. Data processing and visualization were carried out using the *FactoMineR* (version 2.8) and *factoextra* (version 1.0.7) packages in RStudio (version 2025.04.1+524). This multivariate approach enabled the identification of clusters and patterns among variables, and the generated biplots provided clearer insights into the correlations between antioxidant activity and the levels of bioactive compounds.

## 4. Conclusions

This study provides valuable insight into the phytochemical composition, nutritional richness, and antimicrobial potential of *Z. officinale* and *P. nigrum*, two spices widely used in both culinary and traditional medicine. Through a combination of spectrophotometric assays, UPLC-HRMS profiling, mineral analysis, antimicrobial testing, and multivariate statistics, we highlighted the notable bioactivity of both plants.

*Z. officinale* stood out for its strong bacteriostatic activity at relatively low concentrations, especially against *Staphylococcus aureus* and *Salmonella*, likely driven by phenolic compounds such as 6-gingerol, shogaol, and cinnamic acid. On the other hand, *P. nigrum* showed broader antimicrobial effects and richer mineral content particularly in calcium, iron, magnesium, zinc, and manganese, alongside high levels of piperine, a compound known for its antimicrobial and metabolic benefits. These distinct profiles emphasize how each plant contributes uniquely to nutrition and microbial control.

Statistical analyses (PCA and correlation matrices) further supported the central role of polyphenols and flavonoids in antioxidant and antibacterial responses, while also revealing possible interactions with mineral components. This underlines the importance of assessing both chemical and functional properties when evaluating medicinal plants. Beyond their demonstrated bioactivities, these spices show great potential as natural preservatives and as components of functional foods or supplements, especially in immune support and post-infection recovery contexts, such as after COVID-19.

Moving forward, further in vivo studies and exploration of synergistic effects in complex food systems are needed, along with efforts to standardize extraction methods for better reproducibility. Overall, this work adds to the growing evidence supporting the multifunctional value of traditional spices in health, nutrition, and sustainable food preservation.

## Figures and Tables

**Table 1:** Total phenolic content (TPC), total flavonoid content (TFC), total condensed tannins (TCT), and antioxidant activity (CI_50_ DPPH) of *Z. officinale* and *P. nigrum* extracts.

	Extract	TPC (µg GAE/mg Extract)	TFC (μg QE/mg Extract)	TCT (μg CE/mg Extract)	CI_50_ DPPH (μg/mL)
***Z. officinale***	Aqueous	4.105 ± 0.010	14.150 ± 0.182	0.802 ± 0.001	86.290 ± 0.170
	Ethanol	75.694 ± 0.097	114.150 ± 0.79	2.407 ± 0.100	32.620 ± 0.055
	Methanol	54.523 ± 0.331	82.674 ± 0.500	4.654 ± 0.200	47.196 ±0.129
***P. nigrum***	Aqueous	11.512 ± 0.100	2.407± 0.040	5.620 ± 0.130	219.366 ± 0.513
	Ethanol	20.93 ± 0.213	5.617 ± 0.060	21.170 ± 0.300	182.171 ± 0.391
	Methanol	16.467 ± 0.198	2.086 ± 0.010	12.040 ± 0.040	175.527 ± 0.366

GAE—gallic acid equivalent; QE—quercetin equivalent; CE—catechin equivalent; CI_50_—concentration required to inhibit 50% of DPPH radicals.

**Table 2:** The bioactive molecules detected in ethanol extracts of *Z. officinale* and *P. nigrum.* D: Detected, ND: not detected.

Molecule	Chemical Formula	Structure	Retention Time	*Z. officinale*	*P. nigrum*	Bioactivity	Bibliography
6-Gingerol	C17 H26 O4	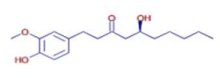	24.61	D	ND	Antioxidant and antiviral	[34]
Shogaol	C11 H14 O3	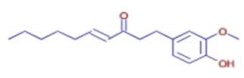	24.61	D	ND	Anticancer	[35]
Cinnamic Acid	C9 H8 O2	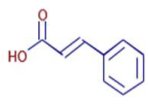	21.49	D	ND	Anticancer and antidiabetic	[36]
5-Carboxyvanillic Acid	C9 H8 O6	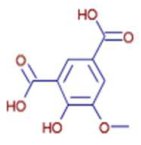	2.59	D	ND	Antioxidant	[37]
Ethyl Cinnamate	C10 H10 O2	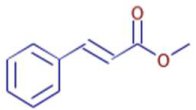	23.72	D	ND	Anti-inflammatory	[38]
Vanillin	C8 H8 O3	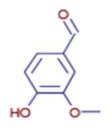	8.46	D	D	Antibacterial	[39]
Piperine	C17 H19 N O3	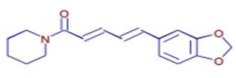	25.37	ND	D	Antibacterial	[40]
Piperanine	C17 H21 N O3	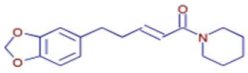	25.37	ND	D	Antimicrobial	[41]
Piperlonguminine	C16 H19 N O3	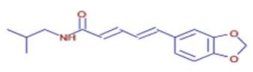	23.63	ND	D	Anti-inflammatory	[42]
Bergenin	C14 H16 O9	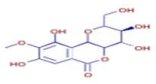	3.90	ND	D	Antioxidant and anti-inflammatory	[43]
Piperonal	C8 H6 O3	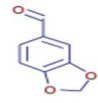	8.72	ND	D	Antiobesity	[44]
Trans-Geranic Acid	C10 H16 O2	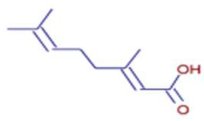	27.18	ND	D	Antimicrobial	[45]
Kanzonol B	C20 H18 O4	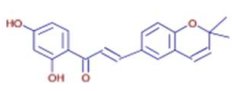	21.77	ND	D	Antibacterial and anti-inflammatory	[46]
Gentisic Acid	C7 H6 O4	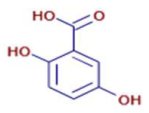	2.72	ND	D	Antioxidant	[47]

**Table 3:** Mineral metal contents in the investigated plants (mg/g dry matter).

mg/g Dry Matter									
	Na	Ca	Mg	K	Fe	Cu	Zn	Mn	B
*Z. officinale*	1.251 ± 0.092	1.430 ± 0.070	2.371 ± 0.131	0.751 ± 0.050	0.171 ± 0.019	0.003 ± 0.001	0.012 ± 0.006	0.191 ± 0.008	0.002 ± 0.001
*P. nigrum*	0.639 ± 0.070	4.160 ± 0.270	1.923 ± 0.180	0.589 ± 0.030	4.030 ± 0.585	0.300 ± 0.033	0.650 ± 0.040	1.760 ± 0.101	0.580 ± 0.080

**Table 4:** Antibacterial activity of *Z. officinale* and *P. nigrum* extracts against *E. coli*, *Salmonella*, and *Staphylococcus* (zone of inhibition in mm).

		Zone of Inhibition (mm)		
Plant	Concentration (mg/mL)	*E. coli*	*Salmonella*	*Staphylococcus*
***Z. officinale***	100	14.00 ± 0.50	7.00 ± 1.00	12.00 ± 0.50
	50	13.00 ± 0.00	-	7.50 ± 0.50
	25	9.00 ± 0.00	-	-
	12.5	7.00 ± 0.00	-	-
	6.25	-		-
***P. nigrum***	100	15.00 ± 1.50	12.50 ± 0.50	14.00 ± 1.00
	50	9.00 ± 0.05	11.00 ± 0.00	11.50 ± 0.50
	25	8.00 ± 0.00	Trace	10.00 ± 0.00
	12.5	7.00 ± 0.00	-	7.50 ± 0.00
	6.25	-	-	-
*Ciprofloxacin (5 µg)*		28.00 ± 2.00		
*Gentamicin (10 µg)*			23.00 ± 1.00	
*Oxacillin (1 µg)*				24.00 ± 1.00
Water distilled	100	-	-	-
	50	-	-	-
	25	-	-	-
	12.5	-	-	-
	6.25	-	-	-

**Table 5:** Minimum inhibitory concentration (MIC) of the aqueous extracts against selected bacterial strains.

Aqueous Extract	MIC (µg/mL)		
	*E. coli*	*Salmonella*	*Staphylococcus*
*Z. officinale*	62.5	3.91	3.91
*P. nigrum*	125	62.5	31.25

## Data Availability

The data generated and analyzed during this work are available from the corresponding author on request.
