# Correction: Aicha et al. Integrated Phytochemical Profiling, UPLC-HRMS Characterization, and Bioactivity Evaluation of *Zingiber officinale* and *Piper nigrum*. *Int. J. Mol. Sci.* 2025, *26*, 7782

**DOI:** 10.3390/ijms27219546

**Published:** 2026-10-26

**Authors:** Aicha Boubker, Abdelmoula El Ouardi, Taha El Kamli, Mohammed Kaicer, Faouzi Kichou, Khaoula Errafii, Adnane El Hamidi, Rachid Ben Aakame, Aicha Sifou

**Affiliations:** 1 Laboratory of Materials, Nanotechnologies and Environment, Faculty of Sciences, Mohammed V University in Rabat, BP: 1014, Ibn Battouta Avenue, Rabat 10000, Morocco; aicha.boubker@um5r.ac.ma (A.B.); a.elhamidi@um5r.ac.ma (A.E.H.); 2 Department of Microbiology of Water, Food and Environment, National Institute of Hygiene, BP: 769, Agdal, 27, Avenue Ibn Batouta, Rabat 10000, Morocco; abdoelouardi@yahoo.fr; 3 Laboratory of Anti-Doping Control, Hassan II Institute of Agronomic and Veterinary, BP: 6202, Madinat Al Irfane, Rabat 10101, Morocco; elkamlit@yahoo.fr; 4 Laboratory of Analysis, Geometry and Applications, Systems and Optimization, Ibn Tofail University, Campus of University, BP: 242, Kenitra 14000, Morocco; mohammed.kaicer@uit.ac.ma; 5 Department of Pathology and Veterinary Public Health, Hassan II Institute of Agronomic and Veterinary, BP: 6202, Madinat Al Irfane, Rabat 10101, Morocco; kichou.faouzi@gmail.com; 6 African Genom, University Mohammed VI Polytechnic Center, BP: 660, Hay Moulay Rachid, Benguerir 43150, Morocco; khaoula.errafii@um6p.ma; 7 Laboratory of Food Toxicology, National Institute of Hygiene, BP: 769, Agdal, 27, Avenue Ibn Batouta, Rabat 10000, Morocco; benakame@yahoo.fr

After the publication of the article [1], one of the referenced manuscripts was found to have been retracted. To enhance the quality and scientific rigor of the manuscript, the author decided to remove the previous reference number 23 [2] from the reference list and replace the original reference with [3]. The correct paragraph in Section 2.2 appears below.

“Significant variations in the profiles of bioactive compounds, which are correlated with the biological activities of *Z. officinale* and *P. nigrum*, were found by comparing their phytochemical analyses. Phenolic substances, including 6-gingerol, shogaol, cinnamic acid, and 5-carboxyvanillic acid, which exhibit significant antioxidant activity and potential anticancer effects, were discovered to be especially abundant in *Z. officinale*. These findings are consistent with other research showing the phenolics generated from ginger can scavenge radicals and may have a function in preventing disease.”

The authors state that the scientific conclusions are unaffected. This correction was approved by the Academic Editor. The original publication has also been updated.

## References

[1] Boubker A., El Ouardi A., El Kamli T., Kaicer M., Kichou F., Errafii K., El Hamidi A., Ben Aakame R., Sifou A. (2025). Integrated Phytochemical Profiling, UPLC-HRMS Characterization, and Bioactivity Evaluation of *Zingiber officinale* and *Piper nigrum*. Int. J. Mol. Sci..

[2] Anh Nga N.T., Sathiyavimal S., Al-Humaid L.A., Al-Dahmash N.D., Lee J., Barathi S., Jhanani G.K. (2023). Deciphering the Anticancer, Anti-Inflammatory and Antioxidant Potential of Ti Nanoparticles Fabricated Using Zingiber officinale. Environ. Res..

[3] Shaukat M.N., Nazir A., Fallico B. (2023). Ginger Bioactives: A Comprehensive Review of Health Benefits and Potential Food Applications. Antioxidants.

